# Investigating antimicrobial resistance genes in Kenya, Uganda and Tanzania cattle using metagenomics

**DOI:** 10.7717/peerj.17181

**Published:** 2024-04-22

**Authors:** Kauthar M. Omar, George L. Kitundu, Adijat O. Jimoh, Dorcus N. Namikelwa, Felix M. Lisso, Abiola A. Babajide, Seun E. Olufemi, Olaitan I. Awe

**Affiliations:** 1 Department of Biochemistry and Biotechnology, School of Pure and Applied Sciences, Pwani University, Kilifi, Kenya; 2 Division of Immunology, Department of Pathology, Institute of Infectious Disease and Molecular Medicine, University of Cape Town, Cape Town, South Africa; 3 Genetics, Genomics and Bioinformatics Department, National Biotechnology Development Agency, Abuja, Nigeria; 4 Department of Data Management, Modelling and Geo-Information Unit, International Centre of Insect Physiology and Ecology, Nairobi, Kenya; 5 South African National Bioinformatics Institute, University of the Western Cape, Cape Town, South Africa; 6 Department of Biochemistry, Ladoke Akintola University of Technology, Ogbomoso, Nigeria; 7 African Society for Bioinformatics and Computational Biology, Cape Town, South Africa; 8 Department of Computer Science, University of Ibadan, Ibadan, Oyo State, Nigeria

**Keywords:** Antimicrobial resistance, Cattle, Metagenomics, Resistomes, East Africa, Antimicrobial Resistance Genes (ARGs)

## Abstract

Antimicrobial resistance (AMR) is a growing problem in African cattle production systems, posing a threat to human and animal health and the associated economic value chain. However, there is a poor understanding of the resistomes in small-holder cattle breeds in East African countries. This study aims to examine the distribution of antimicrobial resistance genes (ARGs) in Kenya, Tanzania, and Uganda cattle using a metagenomics approach. We used the SqueezeMeta-Abricate (assembly-based) pipeline to detect ARGs and benchmarked this approach using the Centifuge-AMRplusplus (read-based) pipeline to evaluate its efficiency. Our findings reveal a significant number of ARGs of critical medical and economic importance in all three countries, including resistance to drugs of last resort such as carbapenems, suggesting the presence of highly virulent and antibiotic-resistant bacterial pathogens (ESKAPE) circulating in East Africa. Shared ARGs such as aph(6)-id (aminoglycoside phosphotransferase), tet (tetracycline resistance gene), sul2 (sulfonamide resistance gene) and cfxA_gen (betalactamase gene) were detected. Assembly-based methods revealed fewer ARGs compared to read-based methods, indicating the sensitivity and specificity of read-based methods in resistome characterization. Our findings call for further surveillance to estimate the intensity of the antibiotic resistance problem and wider resistome classification. Effective management of livestock and antibiotic consumption is crucial in minimizing antimicrobial resistance and maximizing productivity, making these findings relevant to stakeholders, agriculturists, and veterinarians in East Africa and Africa at large.

## Introduction

According to the World Health Organization, antimicrobials are used for the prevention and/or treatment of infections in animals, humans, and plants. Antimicrobials include antibiotics, antifungals, and antiparasitics (WHO, 2021). Antimicrobial resistance (AMR) refers to the ability of microorganisms such as bacteria, fungi, parasites and viruses to evolve and become resistant to antibiotics, and when this happens, infections become more difficult to treat, thereby increasing the risk of disease spread, severe illness, and ultimately death (Prestinaci, Pezzotti & Pantosti, 2015). This has become a global health concern worldwide (Donado-Godoy et al., 2015). The emergence of resistance to antibiotics in microorganisms, especially bacteria, is partially due to the existence of resistance genes (Zalewska et al., 2021), and farm animals, such as cattle being the prime reservoirs for these genes (Kim & Ahn, 2022).

Despite the importance of antimicrobials in the livestock sector, with reference to veterinary medicines used for the treatment of animal infections and growth promotion of animals, the uncontrolled, subtherapeutic doses of antibiotics in cattle farms can promote the growth of bacteria that are antibiotic resistant, which in turn affects the changes in microbial metabolism of the animal’s rumen and the global health crisis associated with antibiotic resistance (Maron, Smith & Nachman, 2013; Economou & Gousia, 2015). The transmission of antibiotic resistance genes (ARGs) occurs majorly through conjugation, which refers to horizontal gene transfer that exchanges mobile genetic elements, such as plasmids and transposons that code for ARGs between bacterial species (Redondo-Salvo et al., 2020). Bacteria can also develop antibiotic resistance through chromosomal mutation that empowers them with the ability to withstand high concentrations of antimicrobials from recurrent exposure (Baym et al., 2016). In other cases, they have natural resistance due to the presence of an impermeable cell membrane or lack of an inherent target molecule for the antibiotic to counter against (Mann et al., 2021).

In Africa, reports indicate that the continent has the lowest antimicrobial usage in animals worldwide (World Organisation for Animal Health (OIE), 2020), even though a high incidence of resistance to antimicrobials have been observed in foodborne pathogens isolated from animals and animal products (Van Boeckel et al., 2019). However, there is limited data in East Africa on antimicrobial knowledge, attitudes, and practices (KAP) (Kemp et al., 2021). It has been recorded that the extensive usage of antimicrobials/antibiotics for treatment of dairy cattle infected with certain pathogens encourages the development of resistant strains in such host animals (Vincent et al., 2018). Research shows that in East Africa, AMR is linked to human-animal contact, community transmission of resistant bacteria, and ease of access to cheap antibiotics (Omulo et al., 2015) but there is little information on the source, diversity, as well as the distribution of antimicrobial resistant genes in the most non-cultivable environmental bacterial-pathogens in Africa.

The shown ability to project AMR from genetic sequencing data is a significant leap in resistome surveillance. There have been efforts to model the evolution of viral pathogens like SARS-CoV-2 using genomic sequence data (Awe et al., 2023), HIV-1 evolution in sub-Saharan Africa (Obura et al., 2022), biomarker discovery (Chikwambi et al., 2023; Nyamari et al., 2023; El Abed et al., 2023), malaria/CoVID-19 biomarker discovery (Nzungize et al., 2022), analysis of RNA-seq and ChIP-seq data (Ather et al., 2018), and Ebola Virus comparative genomics (Oluwagbemi & Awe, 2018). Genomics and bioinformatics have been reported to be playing a key role in newborn screening (Wesonga & Awe, 2022) and in agriculture. For instance, there have been computational methods to define gene families and their expression in legumes (Die et al., 2019). High sensitivity, which is the ability to recognize AMR determinants associated with antimicrobial resistance phenotype and high specificity, which is the ability to recognize the absence of AMR determinants in an antimicrobial susceptible phenotype have been observed depending on the bacterial species studied (Hendriksen et al., 2019a; Hendriksen et al., 2019b). One of the methods that can be used in analysing DNA sequences is metagenomics. Metagenomics refers to the study of the structure and function of the total nucleotide sequences which is isolated and analysed from the entire community of micro-organisms present in a sample (Thomas, Gilbert & Meyer, 2012). A notable technique called shotgun metagenomics, studies the genetic content of an environmental sample by extracting and sequencing its microbial community’s total genomic DNA. As a result, this technique yields a substantial overview of the microbiota and allows for the simultaneous investigation of the taxonomic classification and functional aspects of the microbial communities (Kibegwa et al., 2020), as compared to its counterpart, amplicon sequencing approach which only targets a particular region of the community’s genomic DNA. Hence, shotgun metagenomics is the most suitable approach for our study.

According to the East Africa Integration agenda, the East African Community (EAC) aims at transforming the region into a single market (Umulisa, 2020). This means goods and services will be moving across different partner states and that includes the most widely used dairy products from cattle. For milk alone, Eastern Africa is recorded as the first and leading milk-producing region in Africa. It represents 68% of the continent’s milk production with Kenya and Tanzania being amongst the biggest dairy producers in Africa (Bingi & Tondel, 2015). All of this, when integrated into the value chain, creates employment opportunities through businesses done by the movement of products around the member states.

An understanding of circulating resistomes is thus crucial since cattle management becomes quite difficult with disease burden, leading to a reduced production as well as increasing production costs, which threatens the economic growth agenda of the EAC. In this case, it is important to understand the emergence of resistomes within the states implicated to better manage livestock in maximizing production and lowering risk and production costs. In addition to the cost-related benefits of the study, the outcomes will assist stakeholders in a large extent to improve the standardization protocols that control the circulation of cattle-derived products and guide veterinarians on informed decisions on how to efficiently manage clinical cases.

## Methods

### Workflow

#### Data acquisition

Metagenomics data of Kenyan, Ugandan and Tanzanian cattle gut microbiome was obtained from public repositories. The data was obtained using the search method described in Supplementary methods: Data Search. The Kenyan and Ugandan datasets were retrieved from ENA under bioprojects PRJEB28482 and PRJEB20456 respectively, using fasterq-dump from the NCBI SRA Toolkit (https://github.com/ncbi/sra-tools/wiki/01.-Downloading-SRA-Toolkit). All samples from bioprojects PRJEB28482 (Kenya dataset) were used but only six samples (samples ERR1950666 to ERR1950671) from the bioproject PRJEB20456 (Uganda dataset) were used. This was because Uganda dataset had a significantly higher read depth per sample than the Kenya and Tanzania datasets. The grabseqs tool (Taylor, Abbas & Bushman, 2020) was used to retrieve the Tanzania dataset from MG-RAST server (Meyer et al., 2008) under the project mgp81260. Table 1 highlights the total number of samples and size of dataset obtained from each country.

**Table 1:** Metadata describing experimental aspects of the samples obtained from each country.

Country	Size (.gz format)	Sample count	Sample type	Sequencing Platform	Collection date
Kenya	2.6 Gb	47 (single end)	Rumen liquor and fecal	Illumina Miseq	2016
Tanzania	1.6 Gb	36 (single end)	Fecal	Illumina Miseq V3	2015 and 2016
Uganda	23.2 Gb	06 (paired end)	Fecal	Illumina Hiseq X-Ten	2016 and 2017

#### Quality control

Fastqc was used to assess the quality of the raw reads (Andrews, 2010). Multiqc was used to generate an aggregate report for all fastqc reports per country (Ewels et al., 2016). The retrieved reads were all of good quality with a quality score (Q) greater than 20; thus, we saw no need to further trim them to much higher qualities since from Q ≥ 20 is within tolerable limit for Illumina data without interfering with subsequent downstream analyses.

#### Metagenomics data analysis

The initial metagenomics analysis of taxonomic abundance and functional annotation was achieved with SqueezeMeta v1.5.1 (Tamames & Puente-Sánchez, 2019), which is a pipeline that constitutes all the steps from assembly of reads to functional annotation. Megahit was used for assembling (Li et al., 2015). Prinseq was used to remove short contigs (<200 bps) and determine contig statistics (Schmieder & Edwards, 2011). Barrnap was used to predict RNAs (Seemann, 2014). RDP classifier was used to taxonomically classify 16S rRNA sequences (Wang et al., 2007). Aragorn was used for predicting tRNA/tmRNA sequences (Laslett, 2004). Prodigal was used to predict open reading frames (ORFs) (Hyatt et al., 2010). Diamond (Buchfink, Xie & Huson, 2014) was used to search for similarity between eggNOG (Huerta-Cepas et al., 2015), GenBank (Clark et al., 2015), and KEGG (Kanehisa, 2000). Searches for HMM homology were performed using HMMER3 (Eddy, 2009) from the Pfam database (Finn et al., 2015). Using Bowtie2 (Finn et al., 2015), reads were mapped against contigs. Binning was conducted using MaxBin2 and Concoct (Wu, Simmons & Singer, 2015), and Metabat2 (Kang et al., 2019).

#### Detection of antimicrobial resistance genes (ARGs)

ARGs were detected from the assembly output of the SqueezeMeta pipeline with abricate version 1.0.1 (tseemann, 2020) using all its built-in databases which include CARD (Comprehensive Antibiotic Resistance Database) and resfinder, NCBI AMRFinderPlus, ARG-ANNOT, EcOH, MEGARES 2.00 (Feldgarden et al., 2019; McArthur et al., 2013; Bortolaia et al., 2020; Gupta et al., 2014; Donado-Godoy et al., 2015; Ingle et al., 2016), which is the default setting for robustness and sensitivity to ARGs detection.

### Benchmarking

**Figure 1:**
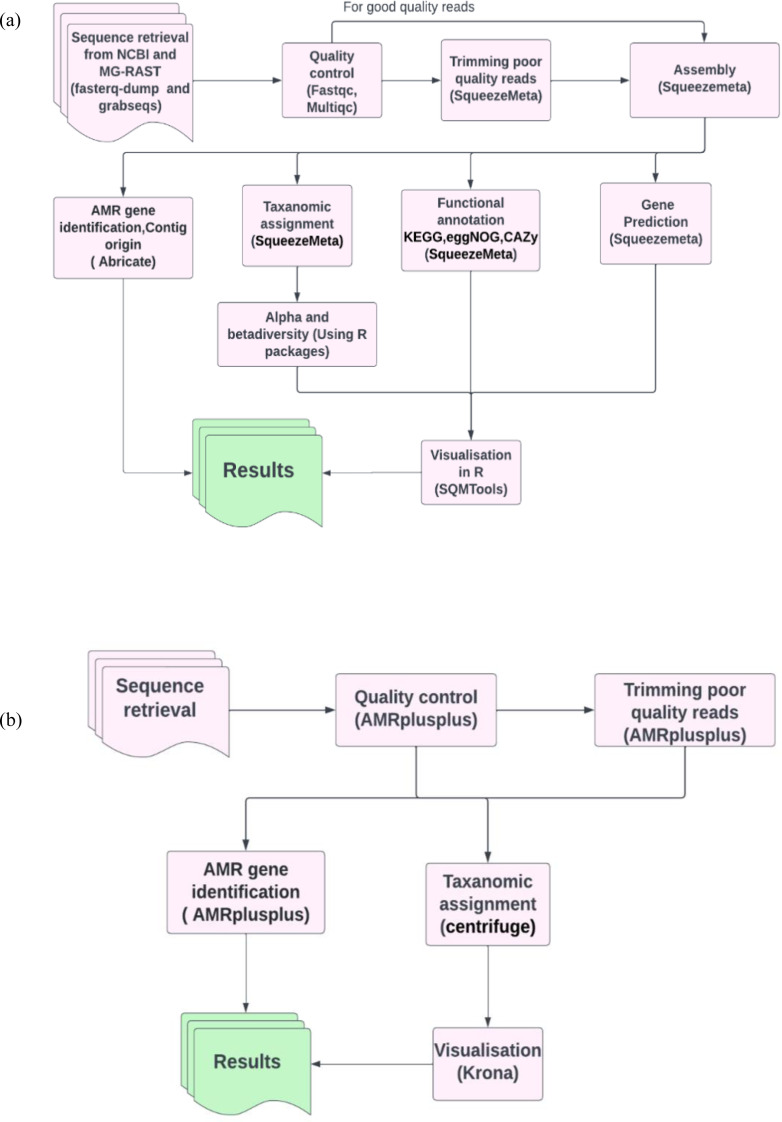
Pictorial representation of the analysis workflows. (A) The assembly based workflow using SqueezeMeta pipeline and abricate. (B) The benchmarking read based workflow using AMRplusplus pipeline, centrifuge and Krona.

To benchmark our analysis workflow (Fig. 1A) containing SqueezeMeta for metagenomics analysis and abricate for ARG’s identification, we compared the efficiency of our workflow (Fig. 1A) with our benchmarking workflow (Fig. 1B). The benchmarking workflow contained AMRplusplus v2.0.2 (Gray & Head, 2008) for resistome identification, centrifuge version 1.0.4 (Kim et al., 2016) for taxonomic assignment and Krona version 2.8.1 (Ondov, Bergman & Phillippy, 2011) for the visualisation of taxonomic profiles. It is worth mentioning that generally (Fig. 1A) workflow was assembly based and (Fig. 1B) workflow was read based. In this way we were also assessing the efficiency of assembly-based methods against read based methods in the identification of ARGs in our study.

### Data visualization

A key stage in data analysis is the interpretation of the obtained results from different sequence analysis tools. This can be done by using plots, graphs, tables etc, depending on the results you have and the question you are trying to answer. Given our study objectives, which aimed to present taxonomic and functional annotations and the visualization of the identified drug classes and ARGs identified, we relied heavily on the use of R and Python programming languages as well as Krona for the visualisation of centrifuge in particular. Portions of this text were previously published as part of a preprint (Omar et al., 2023).

## Results

### Taxonomic microbial abundance and functional annotation

Assessment of taxonomic composition is essential in any metagenomics study. It provides you with the insights needed to draw necessary conclusion with regard to your study. In our case where we were interested in detecting antimicrobial resistance, annotating the potential taxonomies found in our samples aids in giving an idea of the severity of resistance and possible mechanism of transmission of these genes across different bacteria if at all certain strains linked with horizontal gene transfers are to be identified. In this section, we highlight the taxonomic and functional abundance of our analysis samples.

**Figure 2:**
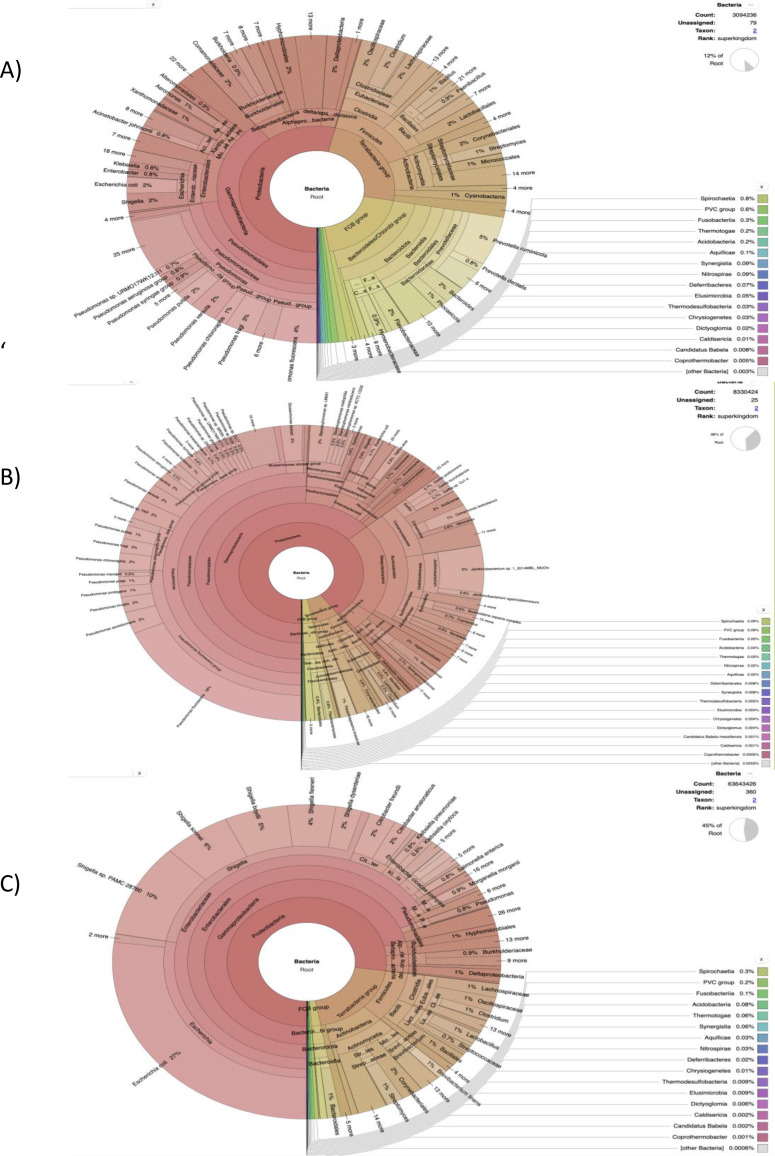
The cattle rumen liquor and fecal excreta metagenome from Kenya, Tanzania and Uganda. Taxonomical assignment was done directly from the metagenomic reads using centrifuge and the taxonomical classification results were imported into Krona for visualization. The hierarchical taxonomic levels are from Kingdom to species from center to the edges respectively but some phyla are within clade groups like Firmicutes phylum in the Tetrabacteria group (clade). (A) Kenyan samples. (B) Tanzanian samples. (C) Ugandan samples.

### Taxonomic Microbial Abundance in Kenya, Tanzania and Uganda

#### Kenya.

Our results highlight some of the details regarding the possible taxonomic composition and functional capacity of microbial communities in samples from each country. In Fig. S1A, a greater proportion of reads were unmapped in the Kenyan dataset. The phyla *Bacteroidetes, Proteobacteria* and *Firmicutes* were observed to be the most abundant in decreasing order, with *Proteobacteria* being most abundant in some samples. All viruses were unclassified here. Similarly, at the family level of prokaryotic abundance, there was a large percentage of unmapped reads as shown in Fig. S2A, produced by SqueezeMeta in the co-assembly mode and the most abundant families were *Prevotellaceae* and *Bacteriodaceae*. *Pseudomonadaceae* was prevalent in a few of the sample reads with a spiked abundance level.

Figure 2A was generated using the custom pipeline (Fig. 1B) to benchmark taxonomic assignment of the metagenomic results obtained from SqueezeMeta. At the phylum level, *Proteobacteria* was generally observed to be most abundant as seen in some of the samples from the SqueezeMeta pipeline. However, at the family level, *Pseudomonadaceae* was observed to be most abundant, which is consistent with what was observed in some samples of the analysis with SqueezeMeta. Here, a better species resolution is observed.

### Tanzania.

In the Tanzanian dataset, *Proteobacteria, Firmicutes* and *Bacteroidetes* are the most abundant phyla (Fig. S1B), a similar observation to that of Kenya. Based on the co-assembly generated by SqueezeMeta, majority of the reads were abundant for the family—*Pseudomonadaceae* in a few samples, followed closely by *Comamonadaceae* and *Oxalobacteraceae* (Fig. S2B). As seen previously with the Kenyan samples, a high proportion of reads were also unmapped. There were unclassified abundances of bacteria and viruses observed in the taxonomy as well.

The benchmark with Centrifuge *i.e.,* a tool used to implement the custom pipeline, was used to assign taxonomic labels to sequence reads from Tanzania (Fig. 2B). The plot also shows species-level assignment as compared to the plot generated from SqueezeMeta. Interestingly, in both plots (Fig. S1A and Fig. 2B), there is a high percentage of the phylum—*Proteobacteria* and unmapped reads as well as a minute level of virus abundance. However, in this plot (Fig. 2B), it details and expands more on the *Proteobacteria* phylum, such as the divisions - *Alphaprotobacteria, Gammaproteobacteria,* and the gram-negative bacteria - *Pseudomonas fluorescens, P. azotoformans, Enterobacteriaceae etc.*

### Uganda.

With the Ugandan dataset (Fig. S1C), there was an uneven distribution of abundances for each phylum in the few samples retrieved from the dataset. The most abundant were *Firmicutes, Proteobacteria* and unmapped or unidentified sequence reads. *Fusobacteria* and *Bacteroides* were only prevalent in a few of them. The most prevalent families observed were *Enterobacteriaceae* and *Oscillospiraceae*.

The benchmark results clearly resolve the most abundant phylum to be *Proteobacteria* (Figs. S1C and S2C). The taxonomic classification in the Fig. 2C is similar at the family level with SqueezeMeta results having the most abundant family as *Enterobacteriaceae*. At species level (Fig. 2C), there is a presence of ESKAPE pathogens: *Enterobacter spp*, *Klebsiella pneumoniae* and *Pseudomonas aeruginosa*.

### Functional annotation of Kenya, Tanzania and Uganda samples

Functional annotation provides an overview of the possible physiological functions that the microbiome might have while they are in the gut environment. In our case this information may be used to understand the possible mechanisms that underlie development of antimicrobial resistance if any. In our study, a total of 15 KEGG orthologue resistance genes were functionally annotated in the Kenyan samples (Fig. S3A). The analyses show that the orthologue—Iron complex outer membrane receptor protein (K02014)—was enriched in a few of the samples, with expression profiles of TPM (transcripts per million) above 3,000, while other genes expressed were below 3,000. A similar trend was observed in the Tanzanian samples (Fig. S3B), with K02014 having high expression levels. On the other hand, there was a disparity of orthologue resistance genes in the Ugandan samples (Fig. S3C), as there was an absence of K02014. However, there were high gene expression profiles in K06147 (ATP-binding cassette, subfamily B bacterial), with TPM levels above 3,000, K07497 (putative transposase) with TPM levels between 2,000 and 3,000 in a few of the samples. All other gene expression profiles were below 2,000.

### Identified AMR drug classes

**Figure 3:**
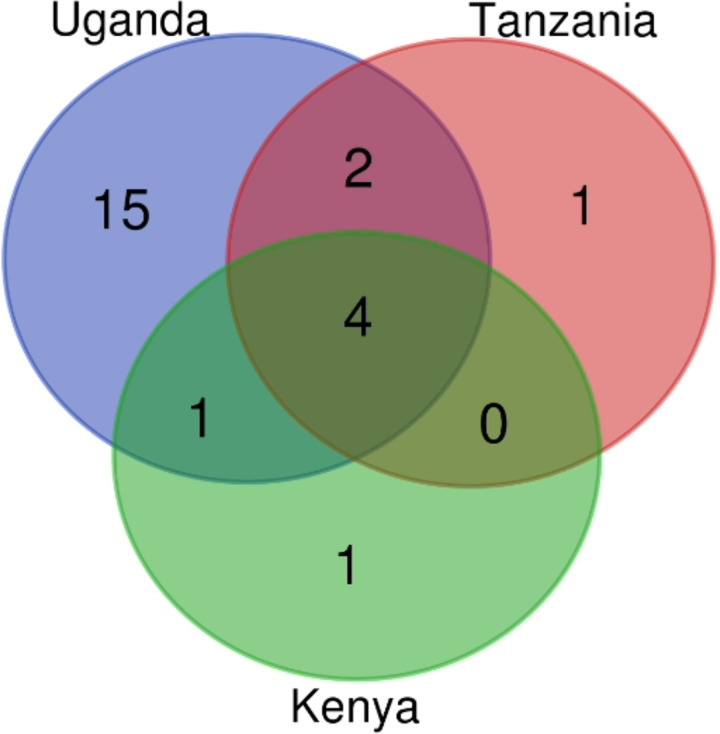
Drug classes count identified in Kenya, Uganda and Tanzania from AMR genes mined from the assembled contigs. The drug classes venn diagram was generated with bioinformatics and evolutionary genomics Venn diagram tool from Abricate’s output. A total of 24 drug classes were identified in the three countries having 22 in circulation in Uganda, seven in Tanzania and six in Kenya.

Understanding the AMR drug classes to which potential AMR genes belong to, helps to provide an overview of the extent of resistance to antimicrobials and the potential threat it poses to public health. In this study, four drug classes of resistance common to Kenya, Uganda and Tanzania were identified from the metagenomic assembled contigs. These were Tetracycline, Beta-Lactam, Sulfonamide and Streptomycin. However, there was a varying prevalence to these drug classes in each of the East African countries. *Tetracycline* resistance was the highest in Kenya and Uganda, with a prevalence of 27% and 24% respectively (Figs. S4A and S4C). Lincosamide resistance was only detected in the Kenyan cattle samples while Streptomycin, Sulfonamide and Spectinomycin resistance was the lowest at 9% (Fig. S4A). Carbapenem resistance was found solely in Tanzanian cattle samples. Additionally in Tanzania, resistance levels were lowest for Chloramphenicol and Trimethoprim (both at 6%). Ugandan samples had 15 unique drug classes, including Cephalosporin, Streptothricin, Quinolone, Nitroimadazole and others (Fig. S4C). The highest number of resistance drug classes was found in Uganda, with a total of 15 (Fig. 3A and Table 2).

On benchmarking our assembly-based approach of ARGs identification with the read-based method in AMRplusplus, we identified a broader spectrum of drug classes since the drug classes belonged to ARGs that were directly mined from the reads rather than the assembly (which does not assemble all reads thus leaving out some information). The number of drug classes identified from reads in the 3 countries were reported to be 59. Uganda has all 59 drug classes with 11 unique to Uganda. Tanzania and Kenya had 44 drug classes each (Fig. S5 and Table S1).

### Identified AMR genes

**Figure 4:**
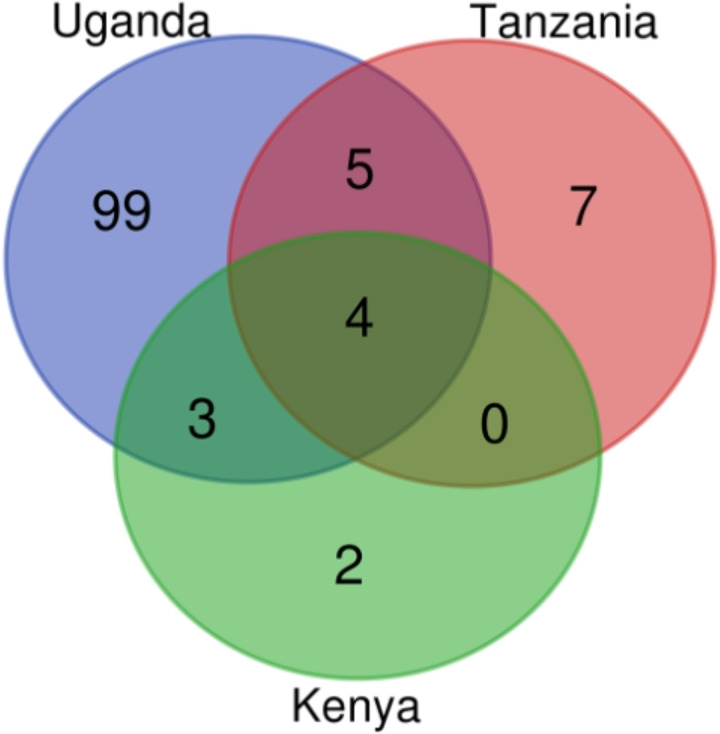
AMR genes count in Kenya, Uganda and Tanzania mined from assembled contigs. The Venn diagram was produced using the bioinformatics and evolutionary genomics Venn diagram tool from Abricate’s output. In total, 120 AMR genes were identified in the three countries, with 111 in Uganda, 16 in Tanzania, and nine in Kenya.

AMR genes in the microbiome pose a possible threat of unmanageable bacterial infections when the bacteria have virulence factors or acquires virulence factors. In this study, we evaluated the possibility of antibiotic resistance in the case of an infection by identifying AMR genes. The Ugandan dataset had the highest number of AMR genes detected from the assembly, totalling to 111 genes (Fig. 4 and Table 3). The common AMR genes identified in the three countries studied are *aph(6)-id* (aminoglycoside phosphotransferase), *tet* (tetracycline resistance gene), *sul2* (sulfonamide resistance gene) and *cfxA_gen* (betalactamase gene). The two AMR genes unique to Kenya include *cfxA6* class A beta (lactamase) and *aadA27* (streptomycin/spectinomycin resistance gene) (Table 3).

**Table 2:** Drug classes in which resistance was identified in Kenya, Uganda and Tanzania mined from assembled contigs.

Country’s name	Count	ARGs drug class
Kenya Tanzania Uganda	4	SULFONAMIDE BETA-LACTAM TETRACYCLINE STREPTOMYCIN
Tanzania Uganda	2	TRIMETHOPRIM CHLORAMPHENICOL;FLORFENICOL
Kenya Uganda	1	LINCOSAMIDE
Uganda	15	ERYTHROMYCIN AMIKACIN;KANAMYCIN NITROIMIDAZOLE AMINOGLYCOSIDE AMIKACIN;KANAMYCIN;QUINOLONE;TOBRAMYCIN STREPTOTHRICIN RESISTANCE VANCOMYCIN QUINOLONE PHENICOL;QUINOLONE CHLORAMPHENICOL CEPHALOSPORIN MACROLIDE FOSFOMYCIN LINCOSAMIDE;STREPTOGRAMIN
Tanzania	1	CARBAPENEM
Kenya	1	SPECTINOMYCIN;STREPTOMYCIN

**Table 3:** AMR genes present in Kenya, Uganda and Tanzania mined from assembled contigs.

Country’s Name	Count	ARGs
Kenya Tanzania Uganda	4	aph(6)-Id tet(40) sul2 cfxA_gen
Kenya Uganda	3	tet(O) lnu(C) tet(W)
Tanzania Uganda	5	sul1 aadA1 aadS tet(A) aph(3″)-Ib
Kenya	2	cfxA6 aadA27
Tanzania	7	floR2 blaTHIN-B blaRm3 tet(G) aadA11 aadA4 dfrB1
Uganda	99	aadA2 dfrA15 tet(44) blaOXY-1-1 blaEC-13 ere(D) fosA4 erm(B) aac(6′)-Ib-cr4 tet(33) erm(Q) msr(D) cmlA1 tet(D) tet(X) mph(B) cfxA2 str dfrA14 lnu(B) vanR-O blaOXA-347 blaEC-5 hugA qnrS1 cmx blaHER-3 tet(S) fosA qnrD1 ant(6)-Ia mef(A) lsa(E) dfrF qnrB6 blaOKP-A-11 tet(M) tet(K) blaOXY-3-1 erm(C) oqxB9 dfrA8 catA14 sul3 tetA(P) blaSED tet(B) erm(F) oqxA10 blaLAP-2 sat2_gen aadA9 tet(32) lnu(AN2) dfrA17 dfrG aad9 fosA8 dfrA23 oqxB20 blaACI-1 catA2 dfrA1 fosA5 blaEC-18 fosA7 aph(3′)-IIIa blaCMY-48 erm(G) qnrB2 tet(Z) qnrB62 tet(L) fosA_gen ant(6)-Ib mef(En2) dfrA12 qnrS13 aadA5 tetB(P) erm(X) dfrA27 blaACT-41 sat4 floR aadE tet(H) blaCMY-157 mph(A) nimE blaCTX-M-152 blaEC-15 dfrA5 blaCMY-151 GENE oqxA9 blaTEM-1 tet(Q) fosA6

A total of 1,425 AMR genes were identified from the reads as a benchmark of the assembly-based approach. Uganda had a total of 1,420 ARGs in circulation, 965 of which were unique to Uganda, while Tanzania had 403 AMR genes in circulation with 3 unique to itself. Kenya had 353 genes in circulation which were also found in either Uganda or Tanzania (Fig. S6 and Table S2). There were many genes identified from reads as compared to those mined from assembled contigs. This is because during assembly, assembled contigs exclude some reads which may contain some of the AMR genes.

## Discussion

In our study, we performed metagenomic analysis on public datasets generated from cattle rumen and faecal samples in the three East African countries; Kenya, Tanzania and Uganda. Our data analysis suggests that *Prevotellaceae, Pseudomonadaceae* and *Enterobacteriaceae* are the most abundant families in Kenya, Tanzania and Uganda respectively. These findings agree with previous reports (Kibegwa et al., 2020; Okubo et al., 2018). *Enterobacteriaceae*, the most abundant family which includes *Escherichia coli*, the most abundant species in Uganda, has been associated with acquiring antimicrobial-resistance-genes from other bacteria through horizontal gene transfer, hence acting as reservoirs of AMR genes (Bailey et al., 2010; Carattoli, 2008). In addition to *E. coli*, a higher number of ESKAPE pathogens such as *Enterobacter spp*, *Klebsiella pneumoniae* and *Pseudomonas aeruginosa* were identified in Uganda. Thus, the presence of high abundance of *E. coli* and ESKAPE pathogens could explain the high number of circulating resistomes in Uganda compared to the other East African countries in the study.

In all three countries, Tetracycline antibiotic resistance was observed to be the most prevalent. This may be due to the fact that Tetracycline is the most widely used broad spectrum antibiotic in the East African countries (Kimera et al., 2020). According to Sangeda et al. (2021), the most common antibacterial agents used in Tanzania are aminoglycosides, beta-lactams, quinolones, tetracycline, trimethoprim, sulfonamides, and various antibacterial cocktails, with the tetracycline class topping the list. The aforementioned findings closely align with our study results to a great extent, as we also found similar resistances to the drugs described by Sangeda et al. (2021) in the Tanzania cattle samples. This could be as a result of the all-time culprit, the overuse or incorrect usage of these antibacterial medications.

The read based method further highlighted that aminoglycosides are the common resistance drug class shared by all the three countries followed by tetracyclines. Elfamycins and MLS (macrolides-lincosamides and streptogramins B) resistance was also identified in Kenya and Tanzania using the read based method. These findings were not featured in the assembly-based method, hence highlighting the strength of detecting more AMR genes that read based methods has over assembly-based methods.

The genes identified to be common in the three countries (*aph6-id, tet, sul2, cfxA_gen*) are of public health significance. In several studies, the gene *aph6-id*, which is an aminoglycoside resistance gene, has been linked to multi-drug resistant *E. coli* in cattle farms in Korea (Belaynehe et al., 2017), Jeddah (Yasir et al., 2020) and Pakistan (Ali et al., 2021). This gives an indication of the widespread distribution of this gene and is a critical epidemiological marker that could increase multidrug resistance in other pathogens through horizontal gene transfer. The genes, *tet* and *sul2* enhance resistance to sulfonamides and tetracyclines, which are very important antimicrobial classes according to WHO’s ranking (WHO, 2017). These antibiotics are heavily used in livestock industry in East Africa (Orwa et al., 2017; Sangeda et al., 2021), and over time, the antibiotic residues in beef from cattle may constitute a health risk to humans when consumed. The presence of *cfx* gene, which encodes for cephalosporin resistance indicates that cephalosporin is also one of the most widely used antibiotics in East Africa. Altogether, this suggests the need for approaches that combat the increasing antimicrobial resistance incidences.

Functional annotation was performed using SqueezeMeta pipeline, which generated heat maps of genes identified in the three EAC countries - Kenya, Uganda and Tanzania. Of the 15 annotated KEGG orthologues, four exhibited significantly elevated gene expression profiles corresponding to the sample genes in the three countries. In Kenya and Tanzania, gene profile K02014 - iron complex outer membrane receptor protein gene, was highly expressed for some samples as seen in Figs. S3A and S3B. This functional profile gives survival advantage to the bacteria by helping the bacteria bind to iron-containing molecules in the environment, thus enabling them to live longer (Palmer & Skaar, 2016; Caza & Kronstad, 2013), potentially leading to the transfer of ARGs or acquiring more ARGs through mutations and horizontal gene transfer.

In Uganda, three KEGG orthologues had a significantly high gene expression profile for some samples as seen in Fig. S3C. These include; functional profiles K06147—ATP-Binding cassette subfamily B, K07497—putative transposase and K07483—transposase, in their respective decreasing expression levels in TPM (Transcripts per million).

According to Vigil-Stenman et al. (2017), transposons play a fundamental role in bacterial genome-plasticity and host-adaptation, although their transcriptional activity in the natural bacterial communities has not been largely explored. Thus, the K07497—putative transposase and K07483—transposase functional profiles as per the KEGG orthologue heat map in Fig. S3C, suggests the possibility that the introduction and use of antibiotics triggered the changes in genomic regions (mediated by transposons) of the bacteria in the samples. Changes in these regions might have resulted into resistant genes with significant expression in some of the sample genes collected from Uganda.

The use of short reads sequences posed challenges in assembling some reads into contigs. Lower sequencing depth in the samples for Kenya and Tanzania in the analysis might have contributed to less observations of drug resistance classes compared to Ugandan samples. Moreover, there was not enough gut metagenomic samples available for all the three countries for our analysis. This hampered having solid conclusion on the resistome status in the three countries.

## Conclusions and next steps

Antimicrobial resistance to drugs and other therapeutics is consistently on the rise, as evidenced by the findings of several studies emphasising the numerous routes by which bacteria harbouring AMR genes may be transmitted to humans. Based on the economic value of cattle in Africa, and beef being a common source of protein, we focused on the East African cattle to determine which AMR genes would be found through shotgun metagenomic analysis. Our findings indicate that East African cattle’s gut microbiome has a wide range of antibiotic resistance genes. Thus, the need for the development of new antibiotic drug classes to suppress the effects of bacteria harbouring AMR genes. Furthermore, the potential of transmission from cattle to humans poses a larger threat.

This study gives an overview of the current state of antibiotic resistance in selected East African countries. This includes the identification of a wide range of ARGs, including ARGs linked to multi drug resistance in *E. coli* such as *aph6-id.* It also associates high prevalence of ARGs to the high abundance of *Enterobacteriaceae* and ESKAPE pathogens. This information could serve as a signal to authorities coordinating East African integration, prompting them to modify and/or formulate policies governing antibiotic use and transit of dairy products across the EAC member nations and Africa at large. It is essential for them to recognize that the development agenda has a cost, which involves focusing and incentivizing researchers to conduct more studies on combating antibiotic resistance, to guarantee a prosperous and harmonious integration of EAC.

Our study acts as a pilot in efforts of AMR surveillance using metagenomics samples in the EAC region. Here, we benchmarked assembly-based methods against read-based methods. Based on our two workflows, we noted that read based methods are more robust for the identification of AMR genes. However, this might vary depending on your research questions and design.

## Supplemental Information

10.7717/peerj.17181/supp-1 Supplemental Information 1 Taxonomic profile abundance at Phylum level for the assembled 4contigs The y-axis represents the taxonomic profile abundance of each phylum as a percentage. Taxonomic assignment of the contigs was done by Squeezemeta using the RDP classifier. The contigs were obtained from Squeezemeta using the MEGAHIT assembler. (A) Co- assembled Kenyan samples. (B) Co-assembled Tanzania samples. (C) Sequentially assembled Uganda samples.

10.7717/peerj.17181/supp-2 Supplemental Information 2 Taxonomic abundance plot at family level for the assembled contigs The y-axis represents the taxonomic profile abundance of each family as a percentage. Taxonomic assignment of the contigs was done by Squeezemeta using the RDP classifier. These are the contigs obtained from Squeezemeta using the MEGAHIT assembler. (A) Co-assembled Kenyan samples. (B) Co-assembled Tanzania samples. (C) Sequentially assembled Uganda samples.

10.7717/peerj.17181/supp-3 Supplemental Information 3 KEGG Orthologues describing the functional annotation of the experimental samples The heatmap was produced by SqueezeMeta. The shading of the heat map is such that, the darker the shade of a pathway, the more abundant that pathway is in that particular sample. (A) Kenyan samples. (B) Tanzanian samples. (C) Ugandan samples.

10.7717/peerj.17181/supp-4 Supplemental Information 4 Drug classes composition for AMR genes mined from the assembled contigs The pie chart was generated in R from Abricate’s output selecting only the drug class column. The colour assignment is arbitrary. (A) Kenyan samples. (B) Tanzanian samples. (C) Ugandan samples.

10.7717/peerj.17181/supp-5 Supplemental Information 5 Drug classes identified in Kenya, Uganda and Tanzania from AMR genes mined from reads The Venn diagram was created using the bioinformatics and evolutionary genomics Venn diagram tool from a text file containing AMRplusplus output. The output files were filtered using the Linux command ‘cut‘ to only include the drug class column. A total of 59 distinct drug classes were found in the three countries, Uganda samples had all the 59 identified drug classes while Kenya and Tanzania had 44 out of the 59 drug classes each.

10.7717/peerj.17181/supp-6 Supplemental Information 6 AMR genes identified in Kenya, Uganda and Tanzania that were mined from the reads. The Venn diagram was generated from an AMRplusplus output text file using the bioinformatics and evolutionary genomics venn diagram tool. The result files were filtered The Venn diagram was generated from an AMRplusplus output text file using the bioinformatics and evolutionary genomics venn diagram tool. The result files were filtered using the Linux command ‘cut‘ to only include the column for AMR genes. A total of 1,420 unique AMR genes were found in the three countries, with 965 in Uganda, 403 in Tanzania, and 353 in Kenya.

10.7717/peerj.17181/supp-7 Supplemental Information 7 Drug classes in which resistance was identified in Kenya, Uganda and Tanzania from AMR genes mined from reads The table was created using the bioinformatics and evolutionary genomics Venn diagram tool beneath text findings from an AMRplusplus output text file. The output of AMRplusplus was filtered using the Linux command ‘cut‘ to only include the column for drug class. This table supplements Supplementary Fig 5 by giving names to the number of drug classes shown in the Venn diagram.

10.7717/peerj.17181/supp-8 Supplemental Information 8 AMR genes present in Kenya, Uganda and Tanzania The counts column indicate the number of unique genes mined from reads in a set of countries or country specified. The table was built using the Venn diagram tool for bioinformatics and evolutionary genomics under text findings from an AMRplusplus output text file. The AMRplusplus output was filtered using the Linux command ‘cut‘ to contain only the column for AMR genes. This table supplements Supplementary Fig 6 by naming the AMR genes that occur in the Venn diagram.

10.7717/peerj.17181/supp-9 Supplemental Information 9 Supplementary Methods

## Abbreviations

AMR: Antimicrobial Resistance
ARGs: Antibiotic Resistance Genes
EAC: East African Community
FDR: False Discovery Rate
ESKAPE: *Enterococcus faecium, Staphylococcus aureus, Klebsiella pneumoniae, Acinetobacter baumannii, Pseudomonas aeruginosa,* and *Enterobacter spp*

## Additional Information and Declarations

### Data Availability

The following information was supplied regarding data availability: The data is available at GitHub and Zenodo: - https://github.com/omicscodeathon/amr_cattle
- Dorcus Namikelwa, Kauthar M. Omar, George Kitundu, Olaitan I. Awe, Lisso_, & Johny. (2023). omicscodeathon/amr_cattle: amr_cattle_code (v1.0.0). Zenodo. https://doi.org/10.5281/zenodo.10253044
The sequences are available at GenBank: PRJEB28482 and PRJEB20456. The Tanzanian Dataset is available at MG-RAST: mgp81260.
