# Supplementary figures and images for: Investigating antimicrobial resistance genes in Kenya, Uganda and Tanzania cattle using metagenomics
Source: PeerJ. 2024 Apr 22;12:e17181. doi: 10.7717/peerj.17181 (PMC11044882; doi:10.7717/peerj.17181)

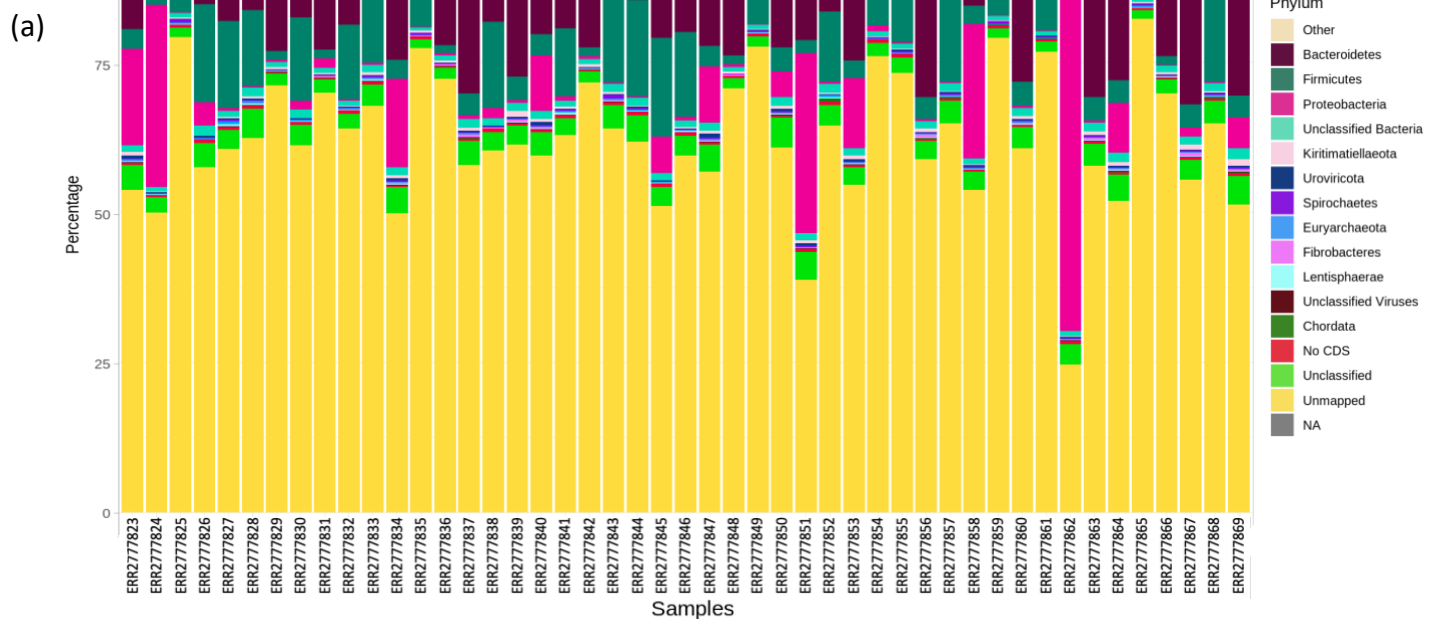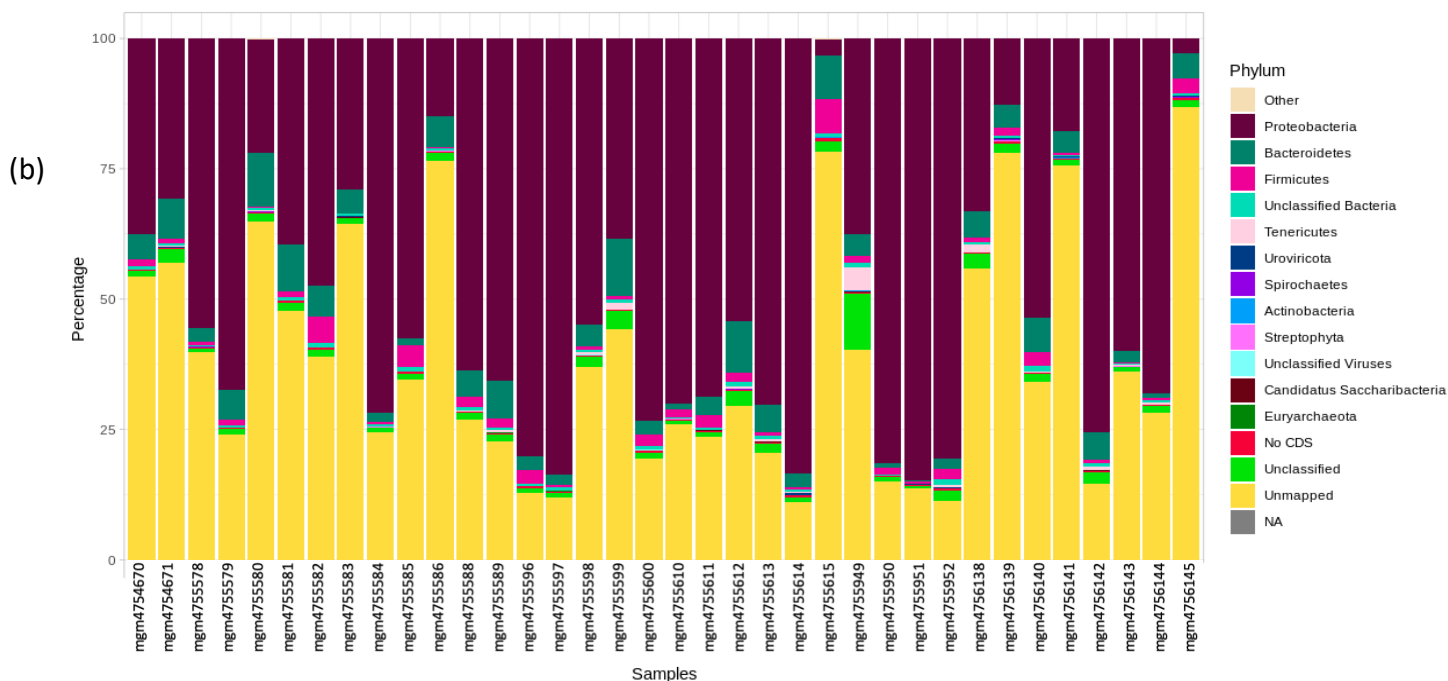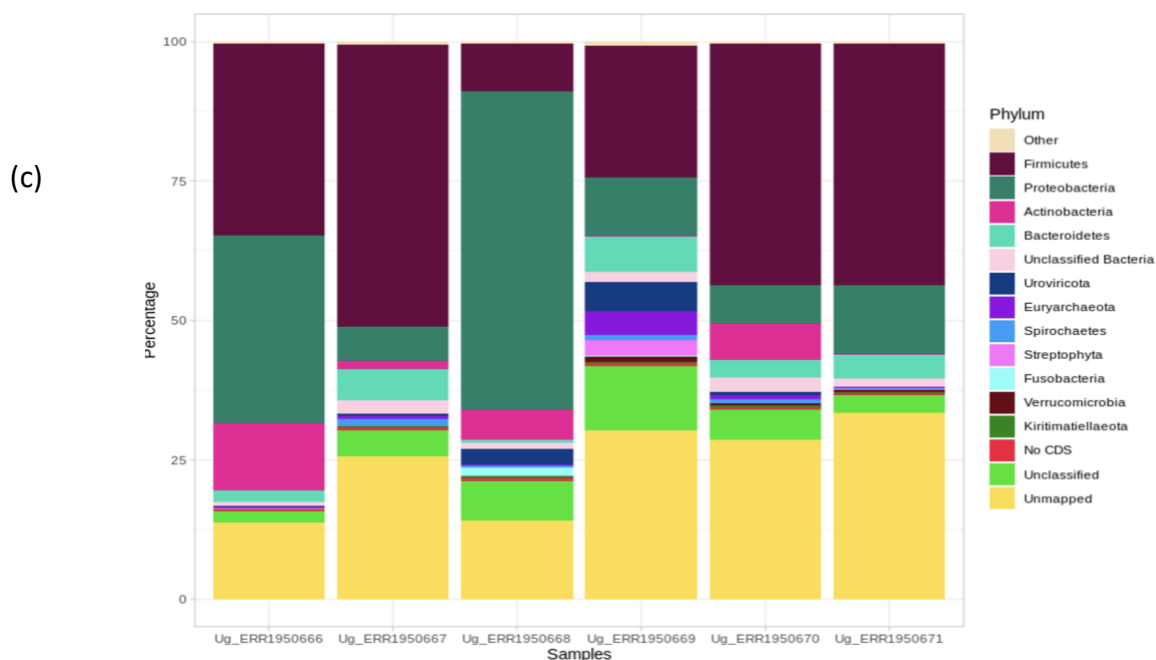

Supplement: Supplemental Information 1 — The y-axis represents the taxonomic profile abundance of each phylum as a percentage. Taxonomic assignment of the contigs was done by Squeezemeta using the RDP classifier. The contigs were obtained from Squeezemeta using the MEGAHIT assembler. (A) Co- assembled Kenyan samples. (B) Co-assembled Tanzania samples. (C) Sequentially assembled Uganda samples. [file peerj-12-17181-s001.pdf]

(a)

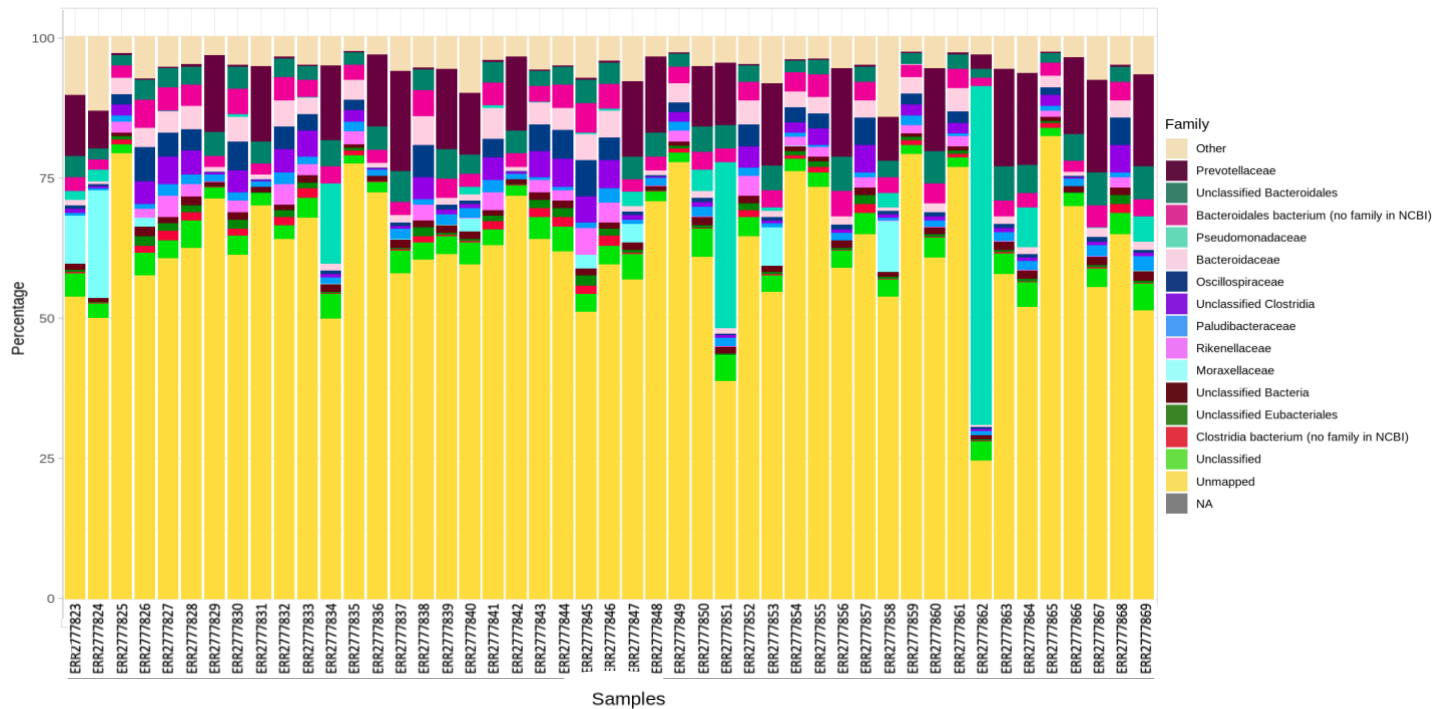

(b)

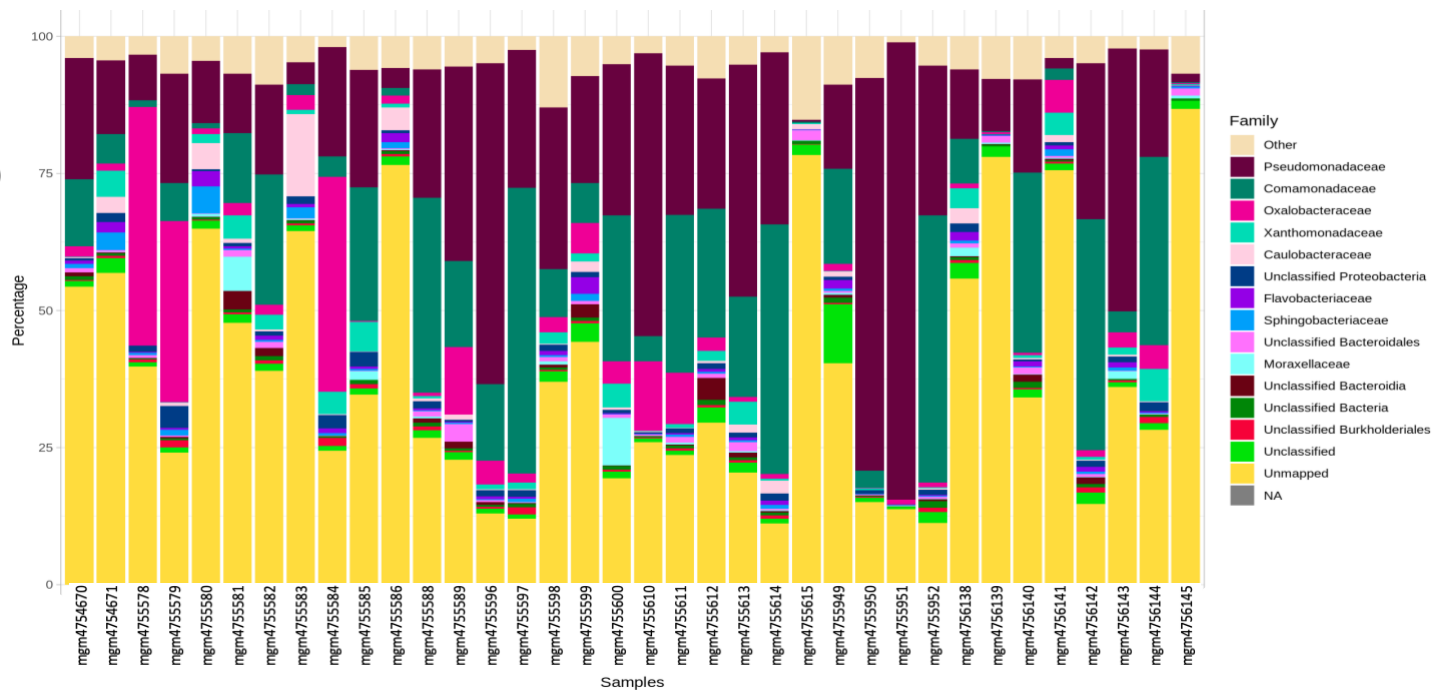

(c)

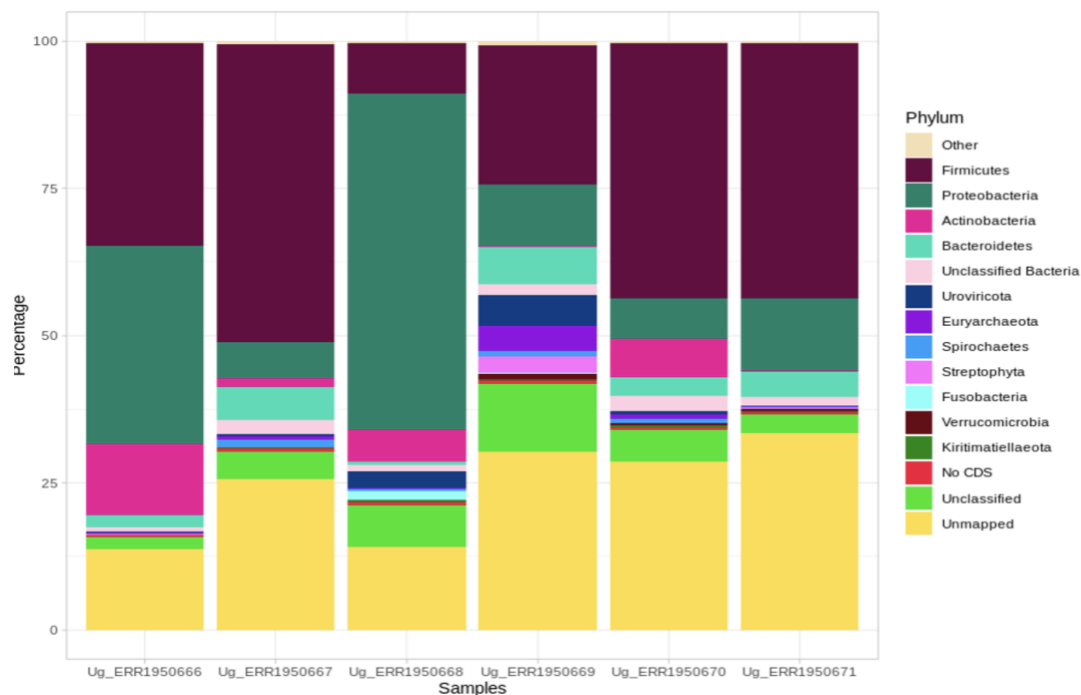

Supplement: Supplemental Information 2 — The y-axis represents the taxonomic profile abundance of each family as a percentage. Taxonomic assignment of the contigs was done by Squeezemeta using the RDP classifier. These are the contigs obtained from Squeezemeta using the MEGAHIT assembler. (A) Co-assembled Kenyan samples. (B) Co-assembled Tanzania samples. (C) Sequentially assembled Uganda samples. [file peerj-12-17181-s002.pdf]

(a)

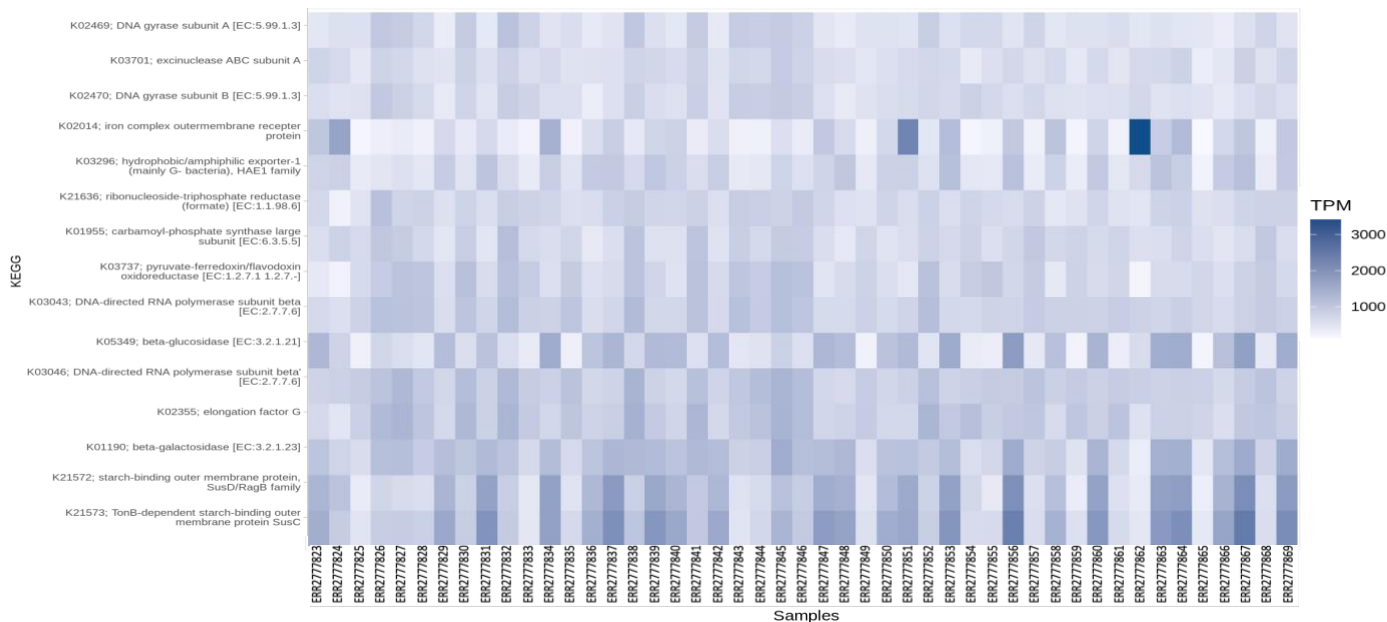

(b)

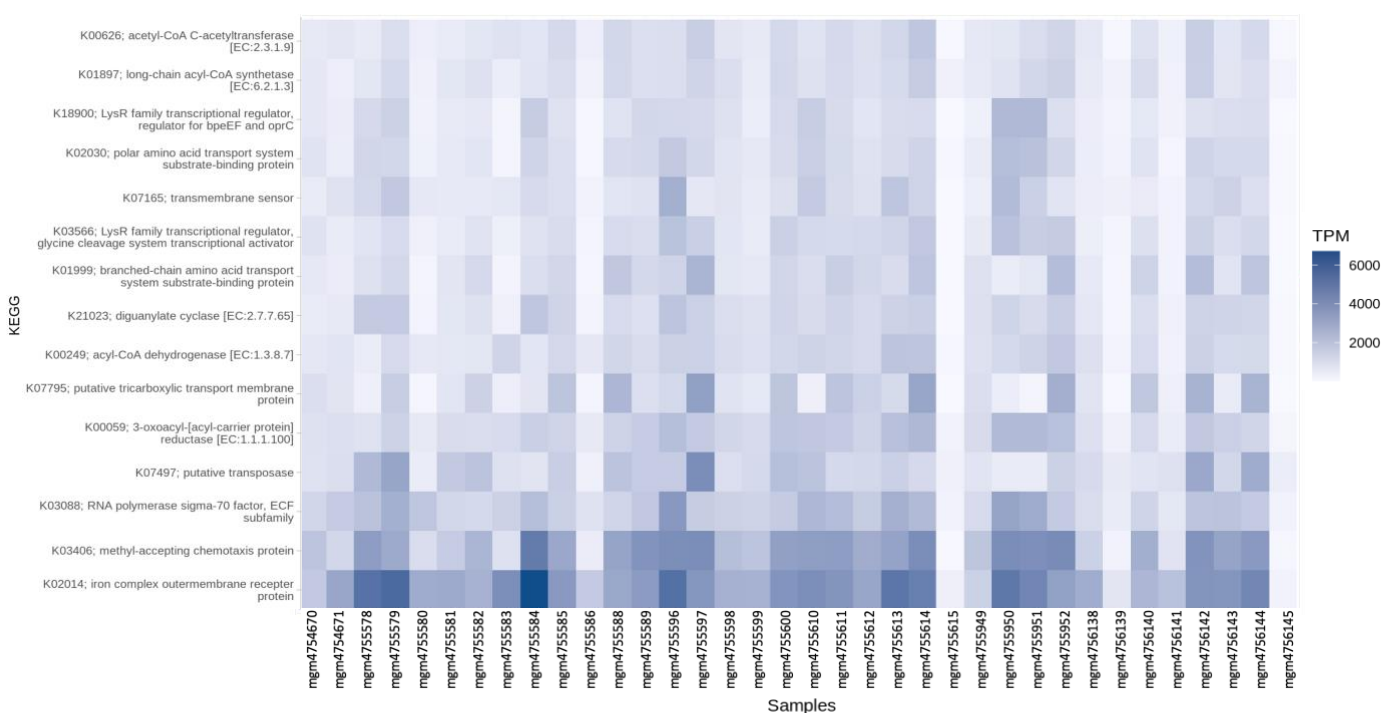

(c)

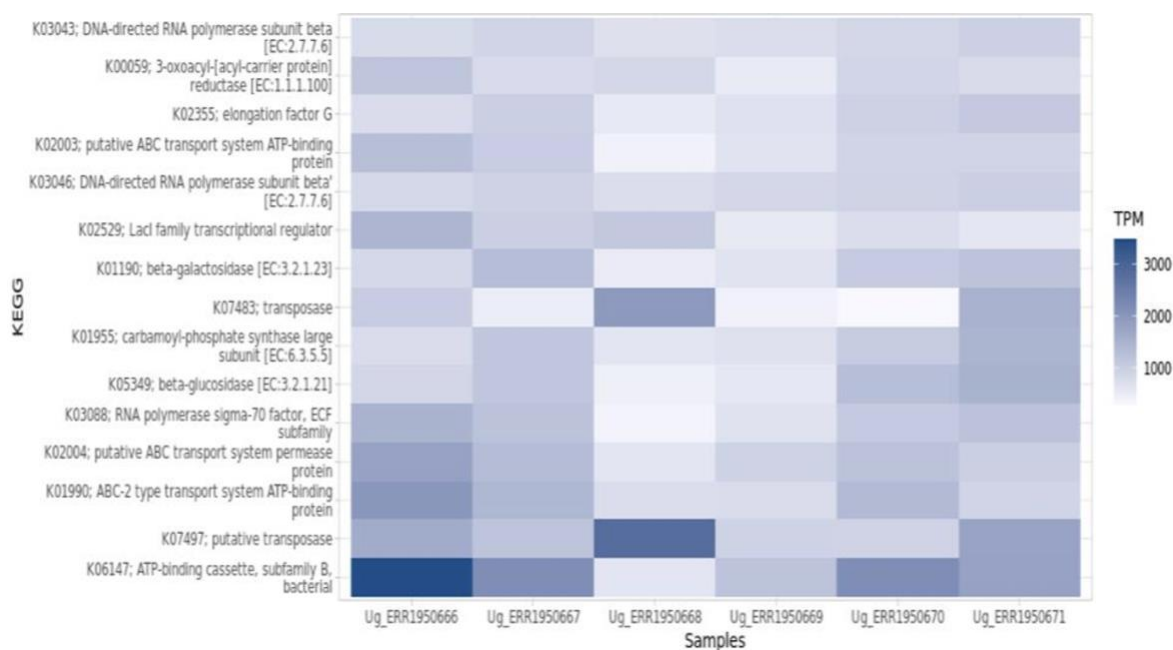

Supplement: Supplemental Information 3 — The heatmap was produced by SqueezeMeta. The shading of the heat map is such that, the darker the shade of a pathway, the more abundant that pathway is in that particular sample. (A) Kenyan samples. (B) Tanzanian samples. (C) Ugandan samples. [file peerj-12-17181-s003.pdf]

(a)

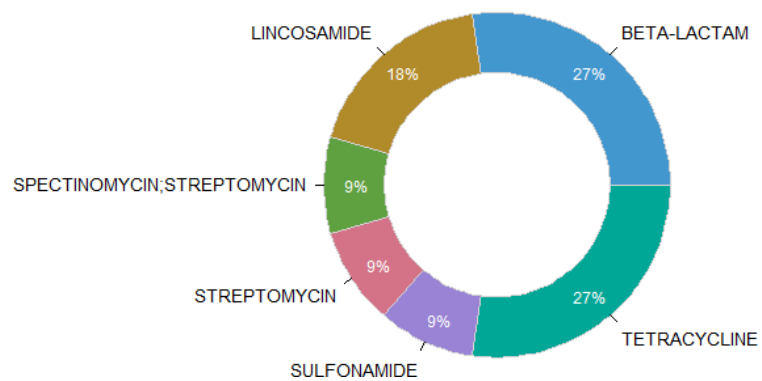

(b)

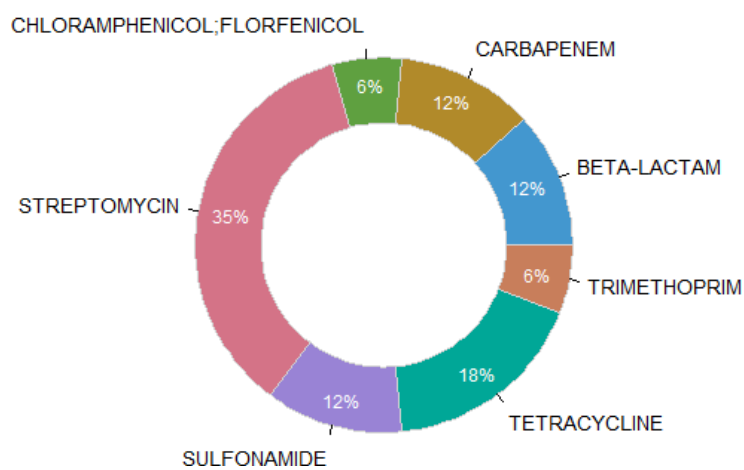

(c)

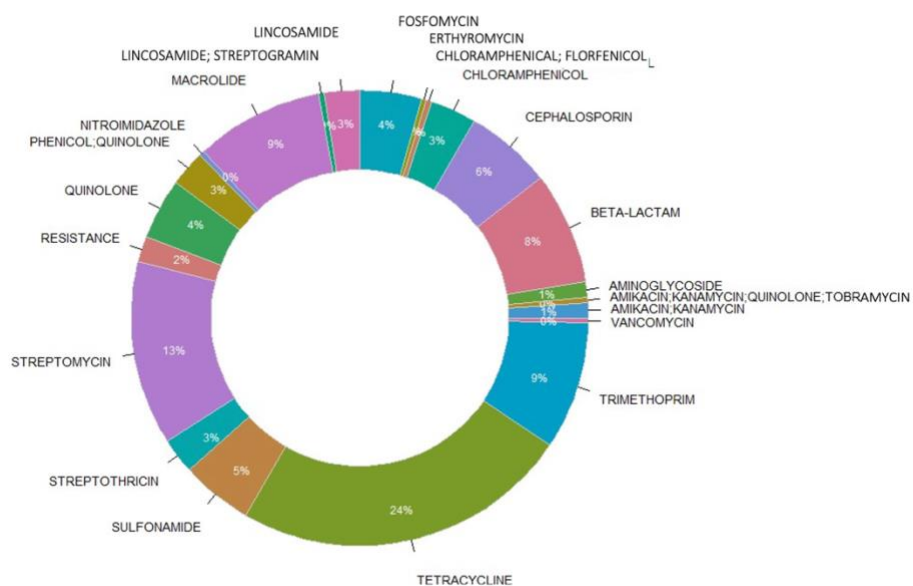

Supplement: Supplemental Information 4 — The pie chart was generated in R from Abricate’s output selecting only the drug class column. The colour assignment is arbitrary. (A) Kenyan samples. (B) Tanzanian samples. (C) Ugandan samples. [file peerj-12-17181-s004.pdf]

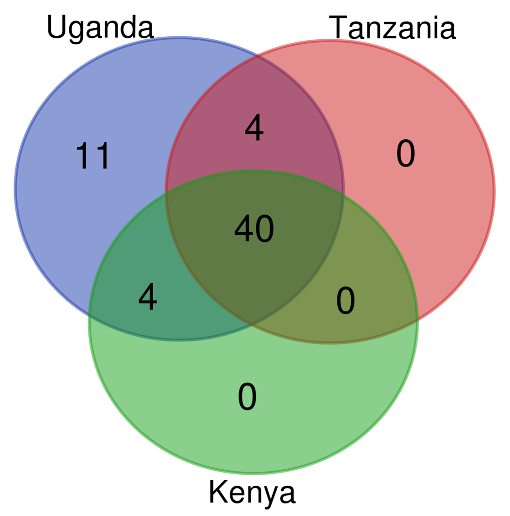

Supplement: Supplemental Information 5 — The Venn diagram was created using the bioinformatics and evolutionary genomics Venn diagram tool from a text file containing AMRplusplus output. The output files were filtered using the Linux command ‘cut‘ to only include the drug class column. A total of 59 distinct drug classes were found in the three countries, Uganda samples had all the 59 identified drug classes while Kenya and Tanzania had 44 out of the 59 drug classes each. [file peerj-12-17181-s005.png]

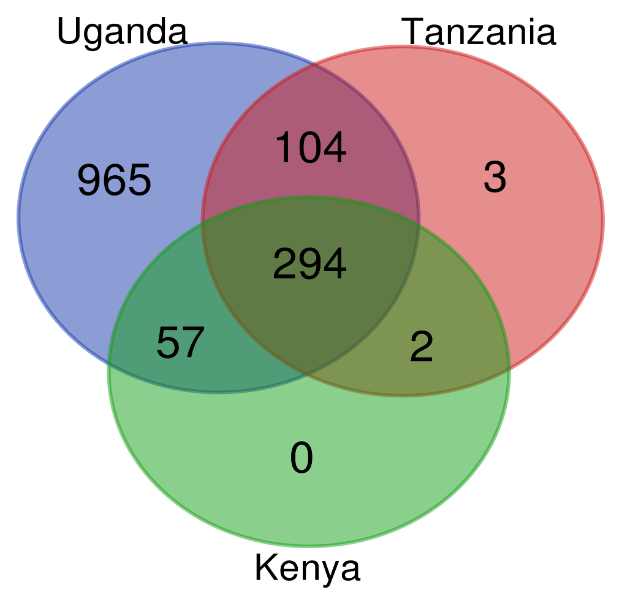

Supplement: Supplemental Information 6 — The Venn diagram was generated from an AMRplusplus output text file using the bioinformatics and evolutionary genomics venn diagram tool. The result files were filtered using the Linux command ‘cut‘ to only include the column for AMR genes. A total of 1,420 unique AMR genes were found in the three countries, with 965 in Uganda, 403 in Tanzania, and 353 in Kenya. [file peerj-12-17181-s006.png]
