# Supplementary material for: Investigating antimicrobial resistance genes in Kenya, Uganda and Tanzania cattle using metagenomics
Source: PeerJ. 2024 Apr 22;12:e17181. doi: 10.7717/peerj.17181 (PMC11044882; doi:10.7717/peerj.17181)
Supplement: Supplemental Information 7 — The table was created using the bioinformatics and evolutionary genomics Venn diagram tool beneath text findings from an AMRplusplus output text file. The output of AMRplusplus was filtered using the Linux command ‘cut‘ to only include the column for drug class. This table supplements Supplementary Fig 5 by giving names to the number of drug classes shown in the Venn diagram. [file peerj-12-17181-s007.docx]

| Country’s Name | Count | ARGs Drug Class |
| --- | --- | --- |
| Kenya Tanzania Uganda | 40 | Aldehyde resistance Glycopeptides Chromium resistance Fosfomycin Phenicol Sulfonamides Fluoroquinolones Lipopeptides Cationic antimicrobial peptides Elfamycins Cadmium resistance Tetracyclines Multi-biocide resistance Acid resistance Bacitracin Drug and biocide resistance Sodium resistance Peroxide resistance Drug and biocide and metal resistance Trimethoprim Pactamycin Rifampin Pleuromutilin Multi-drug resistance Phenolic compound resistance Copper resistance Mercury resistance Aminoglycosides Zinc resistance Acetate resistance MLS betalactams Arsenic resistance Iron resistance Fusidic acid Biocide and metal resistance Nickel resistance Multi-metal resistance Aminocoumarins Oxazolidinone |
| Tanzania Uganda | 4 | Mycobacterium tuberculosis-specific Drug Spiropyrimidinetriones Tellurium resistance Lead resistance |
| Kenya Uganda | 4 | Metronidazole Paraquat resistance Drug and metal resistance Biguanide resistance |
| Uganda | 11 | Cobalt resistance Polyamine resistance Nucleosides Thiopeptides Mupirocin Naphthoquinone Tetracenomycin Quaternary Ammonium Compounds (QACs) resistance Tungsten Resistance Gold resistance Aluminum resistance |
