# Supplementary material for: Investigating antimicrobial resistance genes in Kenya, Uganda and Tanzania cattle using metagenomics
Source: PeerJ. 2024 Apr 22;12:e17181. doi: 10.7717/peerj.17181 (PMC11044882; doi:10.7717/peerj.17181)
Supplement: Supplemental Information 8 — The counts column indicate the number of unique genes mined from reads in a set of countries or country specified. The table was built using the Venn diagram tool for bioinformatics and evolutionary genomics under text findings from an AMRplusplus output text file. The AMRplusplus output was filtered using the Linux command ‘cut‘ to contain only the column for AMR genes. This table supplements Supplementary Fig 6 by naming the AMR genes that occur in the Venn diagram. [file peerj-12-17181-s008.docx]

| Country’s Name | Count | ARGs |
| --- | --- | --- |
| Kenya Tanzania Uganda | 294 | ZNUA TET40 APH3-DPRIME AAC6-PRIME SULII VANC ARSRM TET44 OMPA RPOCR RECG IBPB MEXT GADB COPD RRSC ANT6 CZRA MDTJ MARA YOGI ZUR KPN OMPFB MDTC FABG NHAB PTSL KPNO EMRA SMRA CMLA GLPF PCM EPTB MEFA GYRBA ZUPT PMRAR SODB PSTA COPF CADR NIKR FABI PSTC RUVBM DFRG MEXB COPA LNUC CORB ARSBM MDR23S FLOR NIKD TRIC EMRY RRSH GADE FIEF PBP2 DSBA RRSA MDTO YDEI ZINT FPVA YBTQ OSCA CRP TEHB BACA GADX OMPF YDEP PAREF MODB ACRA TETA DHFR HNS MDTN NFSA EVGA EMRASM CTX EMRR COPC PBP4B TOLC ACRS TETQ CUSB SMET DPSA YHCN YODB COPS MEXC EMHA ACRE NIKB NHAA ROBA EMRB EVGS EEFA MDEA MDTI KDPE BLAEC MDTF CUTE O23S PARC HDEB TETO LPDT FOLP TET39 MSBA BAER MUXB CUSR PITA SODA BHSA FUSA TTGC CFX EMRK NIKE DSBB APH6 MNTH ZNUB ANT2-DPRIME MEXE CUSF SDEY TTGA TETX MDTK ZNTR GYRA TEM OMPR RCNA CATB TET32 TTGR ASR PARE CUTF AMPH FETB DFRA YJCG EMRD CHAA GADW MPHB ACRB MEXN PSTB SOXR EPTA ADEJ CINA EMHC TET16S TETR DNAK ZITB MEXF OMPK36 ZRAR EMRBSM ZNTA NIKC FECD MERD YCHH SULI PHOP PMPM BCR UGD GADC YGJH MDTB TEHA EMRRSM RPOS SILP MDTE YDEO A16S PCOE RRS YAJC UHPT EMRCSM RCNB MDTP CORD IBPA MGTA RRL LPXC TETW RPOCL EMHR OXA MNTP FORMA CORC CUEO SUGE RPOC ICLR PHOR COMR SOXRB ARSCM MDTG NIKA ACRD MLS23S OPMH TUFAB CUEA TTGB MODA CUSC CHR RECGM CUSS AMPC DSBC RPSL MEXW YIEF ARSR ARNA MUXC RUVB SME CUTC OMP36 GLPT CORA ACRR MARR GES FETA CUER MDFA MEXK ASMA SDEB P23S EMHB ACRF PCOD RPOB ANT3-DPRIME P16S YGIW PMRF ZNUC MODC MDTH RCNR MNTR GYRB PH23S CUTA MDTM CAP16S CPXAR MDTA PSTS MURA BAES GADA COPR ZRAS MERP OXYRKP SOXS CUSA HDEA |
| Tanzania Uganda | 104 | TRIA TBTM SILS TETH PHOQ MERE RAHN YEM SSME TET62 TETRM MVRC VANSC MERR1 MUXA CPS ERMF TETC ACTP1 QACH MERA ARSH IMP NCCA FOSK MEXD1 OPRM ERED MEXI PCOC NFXB MERF TETG ALG NALD MERC ARSB SILA VANG RM3 KPNE COPM CARB TOPR NCRC AMPCR MERR NCZA PARR SILR SILC PCOA GYRBZ THINB ARIR COPAM MERR2 TBTA MEFC COPL TRIB RSMA ACN MSRC QACEDELTA1 CZCC CZCA CHRA CPRR ACC QACL FPTA MEXJ PBRA MERT MEXQ THYA COPG APH3-PRIME ESP MERG MEXA CEOB ROSA CATA JOHN TBTB CAT MEXD SILB OPMB TET36 PCOS PARS COPB SILE QEPA TOLCSM DFRB IRLR MRDH QACE OPRN MERB |
| Kenya Tanzania | 2 | ADEK ADEI |
| Kenya Uganda | 57 | RAMA SITB FUSE MOC LPTD MOX APH2-DPRIME ARNT NIMJ MSRE EBR ORN OQXB ADC OMP37 LPXA ABAF KDEA NREB EMMDR PER SITC HOXN FECE SMVA L1 NMPC PCOR KEXD TETB CEPAB OQXA ABEM SDIA ARSDM VANTC SHV PMRBM MEXC1 ACT SMDA CEPH CPHA SMDB CMLB KPNF ZIPB TETY OMPK ADET1 TER VANXA MRPOB TUS ANT9 PHOB BLAA |
| Tanzania | 3 | PSE LCR NPS |
| Uganda | 965 | VMEY TET51 MDE YFMP CRPP CHRR OLED ESTD ABCA TETU SRPB COPV LNUA R39 WUS MYMT LSA CZNC AXC ERMV EFRA CZNA VANXYC IRLS CZCP SALE MEXG SALC ERMR EFM BLACRP YFEC TET63 BRO VANXYL FOSE SFB TRICP ERMO ZIAA BLAL BLAEAM ERMH MMR SPN SPG APMA SMTA TERZ DHFRIII PDRM DHAP MSRD MTRF OTRB CNRC BLAZ OXY VANRG ERM51 OMPD VMEC CMA BLAE RLMA LPSB YCNJ FACT NRSR QNRD CMH HEFA MPHC ADED CRDA PAND SILF KHM SFDC BLASME PLN VGAB HUPE MYX TROR SULX TROB VMEQ ACTS FBPB DPR POM IDER GOLT QACC NPMA MTRR MEFJ FOLC TUNR EFMA RPSE TTGW GOB CFRA TTGV AXY YFEB VGAD ZBMA FUSB VMEJ ACRC CRDS FOSX MEFD FOMB VGBC QNRVC NMTR MCO G2ALT MEXR RPLD MACA GRM FOSD MACB BCII VGAE VANXYE ERMC DFRF VANE LEN PBP2B EATAV KMR LSAC PBP1A TVAB VCAM CNRA MEFF CMEF VANXYN TETA46 PGPA HMB QACF ARSP VARG BEPD ERMT AAC2-PRIME NIMB TET57 TERC TLRCM CTPD PBP2X BEPF YRC RGT NIRC MECD SFO TLYA COPY VMEV PBRR VANSA CZCD RMTA BLAS RANB UL3 AMRA WTPC EBRA NCRAN ARMA CGA SRPCC TRIBP MAL CATT MTRAD CEPA HUPN VMEH CZCB QACJ RMTC CMY EMRE MPHJ TMRB VCEA NORMD FIM LDE SAT RPSA NCRA ARLS ARRA ERM41 TUPC VANTRL VEXH TCR3 MUPB OPTRA FPH EMBC FABV RUB TCMA OKP TTGT CHRI FOSY VATA MEXY BSU SULIII MYRA MFPA IDC SLB SFH TET50 HP VANHB PMRCM TAEA ERM44 ERMA MPHA CDEA MPH QACD ERMX VPH EBRB HPCOPP YKKC KAMB NIMD VANSM VANXYG LMRB VANYG PILQ TRIAP ADEA M1 MEPA GIMA SITABCD TETL CPAA VMLR TET48 RANA VEXC DFRI IRFA BLM FONA TET37 FEZ SCO PEDO BLTD LNUF TERW TETBP BUT MEXX FARR VIM CUTR DES CFRB SPR BLAPAM CMEB VANXD FOSG ADEH FOSU RCP CLBA RMTE SALA VATD MECA ANT4-PRIME LRG MTRA MUS PERR COPZ NCCC BASRS FUSC LNUP FIA SDRM TET58 ACTP CHRE VMEF VANRO TMB MSRF HERA BES ARLR AIOB VANSD VANRD VANK AXYZ VEXF APH9 ADET2 TETA60 GIDB BIC RSA PDC GESB PRC VCRM TETV TET34 MURG VCMA CAU AST PSV NIRB PNGM COPJ EMBA TETE VANSO GRDA TETJ CSP VMEZ ALMG DRRA BLAPLA FMRO BFRA AHPC MERH VANB CORR PENA SRMB LRA TET56 TET30 TRGB TET53 MEXV EDEQ DIM MPHO DFRD AFM POR WALK LMRC MEFB VANRI RAMR ICR ABES CZCR CLBC VANI INIB CMER HMRM VEXA CPT ORR YFEA BLAC ARSA TUPB FRI VAN VMRA DHT NIMF EREB SITA NORA TROC APH4 LAQ RSC CADC AIM ANA LMRP CHRC FURA MEXL OLEC MDR GIL MPHE ARSM NCRBN SRPC FBPA KLAB FEXA YYBT WTPA RMTG NHLF ADEF YBTP ACR3 VATI LPEA MTRC CEOA SGM NIA ADER QNRS NALC HLYD BLT VANRB PST VANXI BCL CMTR VCEC TET42 SULR VGBA YJAA MSRH SALD VCER ALU1P ECV NCCY FARA SDEA OPRA YDDG TCRA BCRA AIOR TROA OTRA AMVA CSA VATF VMED VANWA SRPS CADX QACK AMRB OPMD BC FABK MEXH FOSL OPRZ ERME TRU MSRL ARMR VANSB LPSA TCRZ FUSH NCCB QNRC FOX CATS PBRB TERD CMLV AAK OTRC SIM AIOE TLMB BLA1 INIA ERM30 NCRYN FOSM NCRY CZCCM PBP3 CIA CADA MGRA PBP1 ACTA PCOB VMEK SHN CRDR NIMH MREA LIAFSR FXRA SRT VANYB CATP GIM ZEVA OCH CMX ADEL SRPA PNCA VANRN YFMO EMTA AQU ARST CRD ERM TET54 PEXA RSD1 EFPA MDRL ERMQ CHRB1 VANRA MTRE EMEA CRH KLUC VANWI COPBM CZRB SMB CORT MEXS BAHA PENI EMBB CNRB STA EFRB BEPC NIMI BBI VANA ERM52 SALB VMEI ERM37 NCRB QACR KASA NDH NMCA FOSC DHPS BRP IDSA NOVA YCNK FOSF MPRFD BLAF EIS RICR MPHM ABUO ZNEB BLAR MGT PGBB BLAP SMTB CADD NIMA TET64 IRI VATB QNRE VANWG CPRS MNTA RMTB MPHK CGB MACAB CRCB ALR TLA SEPA NRSD TSNR TET35 PATB NORC TBTR MUPA CME CLS QNRA QACPB CML CZCE APH7-DPRIME FAR1 LLMA PMRG GESC RMTD CRDB MEXZ LNUB YKKCL GYRC VATC OPRJ LUS PERO TETT VANYD NPMB PME FTU ACTR MPHH BLA2 QACAB CHRF PBP5 BAT MMCO LMRS RIBD SULIV TET52 AAC3 TTGE BEPE CMEE CMR NIMC SMFY VANHA VGA PLA VANTG CZNB BLAECM HMRR CNRH TET43 PMRAM CFR EXO ERMS AIOX VANRS HSMR BEPG FOSA TVAA RPLC VANXB IND ALI ARSD CUTO TET59 BEXA LSAD CUEP NIRD GOLS CTPC VANTN NIMK ADEC ERM32 FEXB RPH LPEB CEPS VMEB ADEN MIR VAND ERMD HASF MECC TETB46 CFE TERB WTPB BLE VCEB CFIA PBP4 ARL MPRF MSRA VANTML CMEC ROSB CVI CMLR CMED VMEP RMTF VEXE DMER VGBB ZOG GESA VMEG CTPG VANXO TTGF CLBD MECB CZCS MSRG HPCOPA VANSE ACTPC MCR LSAE GALE ABAQ NIRA BCRB ADEG BJP1 COPK PATA VANRC NRSS TTU ETHR SRPR TAP VATE OLEI PBRT TET33 ADEB BCR1 FABL GRD VANZA BKC ARR VANRE BCRC MCA VANN ERMN VANSN VMB TETD VANL ZEVB KGMB EEFX LHK EMBR VMEU OPRD CKO RDXA TUPA MPHI KMTR ETHA MSHA DFRE MEFE CDSA CBLA BLAB NORM CHRBM PFM CAM MEPB CUID INHA NIME ELM RBPA SED BLTR BLAI LNUG FOSB KMRA BLASGM TET45 BCRCM HUGA NORB ARRB SFC ERMG FRNE LIN ACI IMI INIC CFRC LNUE MECI VEXD VANSG ERMB VANTE NIXA QNRB DHA EVM COPP RLMH MCTB NCCN OPCM FOMA CMEA RPSJ SPM KATG RMTH MPHD NCCH TETS CNRT PBRD ARSHN SDEX POXT ADES CLBB RSD2 VEB GRLB GSHF IBCR KLAC FUSF MTRD ERMU ADEE GRLA NORBD NSHR NCCX TETB60 ERP CEUE VANHD GAR CBP DFRK OLEB TCRB AIOS VMEE VCC GPC MENA TLRC REMB DMEF VEXK PAC TETZ PFR DFRC MEPR TERE CNRR VANYA NCRCN PGSA SFH1 VANSL VANXM VANYF QACG TET38 ERM42 TCRY CARA FARB BMR TETM MPHG RV0678 LMRA QACZ SMR HEFC VANWB ILES TET49 VPOC CSOR CHRB TET31 VANRL FBPC TET55 TRGA CORS MSI LAP EREA VEXB CNRY ROX MYO OMP38 NIMG LMRD CTPV TET47 MPHN FUSD NRSA CIPA BPU PARY CMRA SITD NUOA ARSC VAM ERM50 TETK SPS OHIO LSAB |
